# Deep-Sea Fish Distribution Varies between Seamounts: Results from a Seamount Complex off New Zealand

**DOI:** 10.1371/journal.pone.0036897

**Published:** 2012-06-20

**Authors:** Dianne M. Tracey, Malcolm R. Clark, Owen F. Anderson, Susan W. Kim

**Affiliations:** 1 Deepwater Group, National Institute of Water and Atmospheric Research, Wellington, New Zealand; 2 Flinders Centre for Epidemiology and Biostatistics, Flinders University, South Australia, Australia; National Oceanic and Atmospheric Administration/National Marine Fisheries Service/Southwest Fisheries Science Center, United States of America

## Abstract

Fish species data from a complex of seamounts off New Zealand termed the “Graveyard Seamount Complex’ were analysed to investigate whether fish species composition varied between seamounts. Five seamount features were included in the study, with summit depths ranging from 748–891 m and elevation from 189–352 m. Measures of fish species dominance, rarity, richness, diversity, and similarity were examined. A number of factors were explored to explain variation in species composition, including latitude, water temperature, summit depth, depth at base, elevation, area, slope, and fishing effort. Depth at base and slope relationships were significant with shallow seamounts having high total species richness, and seamounts with a more gradual slope had high mean species richness. Species similarity was modelled and showed that the explanatory variables were driven primarily by summit depth, as well as by the intensity of fishing effort and elevation. The study showed that fish assemblages on seamounts can vary over very small spatial scales, in the order of several km. However, patterns of species similarity and abundance were inconsistent across the seamounts examined, and these results add to a growing literature suggesting that faunal communities on seamounts may be populated from a broad regional species pool, yet show considerable variation on individual seamounts.

## Introduction

Seamounts, knolls, and hills are prominent features of underwater topography in the New Zealand region, with over 800 known from within the Exclusive Economic Zone (EEZ) [1], [2]. Often they are located amongst a complex oceanographic circulation system and they can host diverse and abundant benthic faunal communities. Features with summit depths over about 700 m often have habitat forming stony corals (Order Scleractinia) which cover extensive areas of the summit and upper flanks of the seamounts, with a diverse associated fauna (e.g., [3], [4], [5], [6].Seamounts also host aggregations of commercial fish species, and many are the target of substantial deep-sea fisheries [7], [8], [9].

Seamounts have often been regarded as having elevated endemism relative to other habitats [10], [11], [12]. However, limited sampling has been a problem for determining true levels of endemism [13], and the generality of high endemism has been questioned in recent years [14], [15], [5]. Much of the historical research on seamount biodiversity has focused on benthic invertebrates, many taxa of which potentially have less dispersal capability than fishes through reduced mobility (e.g., sessile corals) or limited larval life spans (e.g., nonplanktotrophic larvae of some gastropods) (see excellent review by Shank [16]). Nevertheless, several studies have defined characters of seamount fishes and documented characteristics which distinguish them from those of the general shelf and slope [17], [18], but there have been few studies examining the variation of fish assemblage composition between seamounts.

Tracey et al. [19] examined fish catches from 10 groups of seamounts around New Zealand, and compared them with fish composition on neighbouring slope areas. They found that species composition was generally similar on seamount and slope, although there were differences between seamount groups in different parts of New Zealand. The study pooled data from seamounts in a cluster or complex, and only examined species composition from individual seamounts within one area. They concluded there were too few data available at the time for a robust analysis, but there was a suggestion of substantial variability between individual seamounts. On a larger geographical scale, Clark et al. [20] compared deep-sea fish fauna on seamounts from nine regions in the North Atlantic and South Pacific Oceans. Fish community composition was found to vary between many of the areas, and there was also considerable variability within areas for several of the datasets. Environmental characteristics of water masses were thought to be important for structuring the fish communities. Nitlitschek et al. [21] examined changes in the composition and abundance of fish species taken during 8 years of fishing for orange roughy (*Hoplostethus atlanticus*) on seamounts off Chile. They found the abundance of bycatch species decreased at more heavily fished sites, and there were indications of differences in species richness, diversity and species assemblage composition between fished areas.

**Figure 1:**
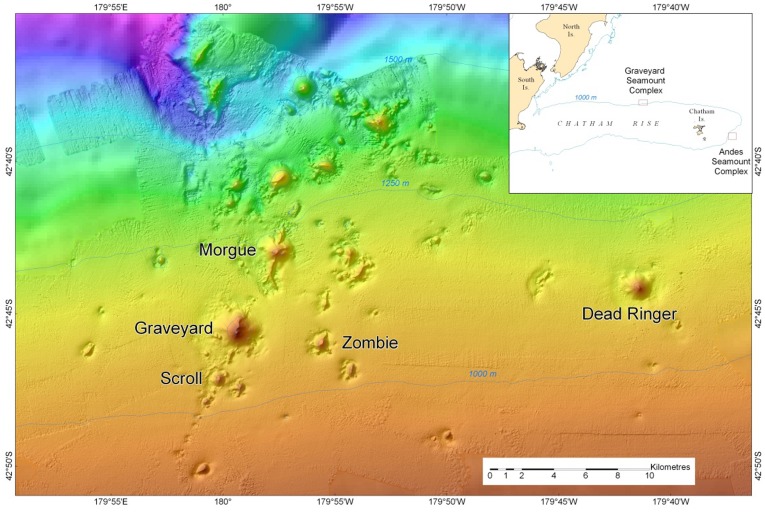
Location of the Graveyard Seamount Complex on the Chatham Rise, east of New Zealand showing location of features for which data were analysed.

In this paper we examine variability between seamounts in a cluster of seamount features east of New Zealand (Figure 1), known as the “Graveyard Seamount Complex” [22], [23]. The features are reasonably closely grouped, and generally similar in size, depth, and elevation. Detailed data from fishery research trawl surveys are examined and the study tests the hypothesis that fish species composition will be similar between seamounts over small spatial scales, and also investigates whether levels of fishing, environmental factors, and physical variables affect species composition or abundance.

## Methods

### Data

The data used for this study came from ten research trawl surveys carried out between 1994 and 2005 to estimate the abundance of orange roughy on the Chatham Rise. All vessels used similar trawl gear, a standard six-panel, rough bottom orange roughy trawl with cut away lower wings, and cod-end mesh size of 100 mm.

Data were extracted for five key seamounts (those with the most research sampling effort, Tables 1 and 2), from the New Zealand Ministry of Fisheries “trawl” database. Information included fish species composition and catch weight from each trawl, as well as data on position, depth, and duration of tow. Only research trawl stations with an acceptable gear performance were used. Lists of taxa were made by individual seamount for teleost fishes, elasmobranchs (sharks, rays, chimaeras, and ghost sharks), and cephalopods. Certain species were excluded (as per Tracey et al. [19]) due to uncertainty in identification, or when the species were predominantly midwater, rather than demersal. Species selected are listed in Appendix S1, with their scientific name, common name, and 3-letter Ministry of Fisheries code (which is used for a number of figures).

**Table 1:** Physical variables and units of the five seamount features of the Graveyard seamount complex examined in this study.

Name	Latitude(S)	Longitude(W)	Summit depth(m)	Base depth(m)	Temp at summit(°C)	Temp at base(°C)	Area (km^2^)	Elevation (m)	Calculated slope (degrees)	Number research tows	Fishing effort (no. of tows)
Morgue	42.72	179.96	890	1200	5.84	4.20	3.1	310	17.3	16	793
Deadringer	42.74	179.69	820	1150	6.31	4.42	2.4	330	26.3	17	800
Graveyard	42.76	179.99	748	1100	6.76	4.70	4.1	352	17.1	73	2034
Zombie	42.77	179.93	891	1080	5.83	4.80	1.1	189	17.7	14	65
Scroll	42.79	180.00	888	1080	5.85	4.82	1.0	192	18.8	22	104

**Table 2:** Number of research trawls by survey and seamount. Column 1 represents the voyage code, e.g., aex9901 =  the 1^st^ voyage on *Amaltal Explorer* in 1999; tan9908 = 8^th^ voyage of *Tangaroa* in 1999.

Survey code	Morgue	Deadringer	Graveyard	Zombie	Scroll
aex9901	3	3	8	2	5
ama0501	0	3	13	1	4
ora0201	0	1	4	3	1
sra9901	0	0	4	0	0
tan0104	3	0	3	0	2
tan0208	0	4	11	4	5
tan9406	3	4	3	2	0
tan9608	1	0	15	0	0
tan9708	1	2	6	0	1
tan9908	5	0	6	2	4
All	16	17	73	14	22

Data on commercial fishing were obtained from Ministry of Fisheries logbooks which have individual trawl information, which enables location to be determined. However, start and finish positions are recorded only to the nearest 1 degree (about 1.8 km or 1 nautical mile) and hence some manual examination of tow sequences and directions was carried out to assign each tow to an individual seamount (as per Clark and Rowden [3]).

**Figure 2:**
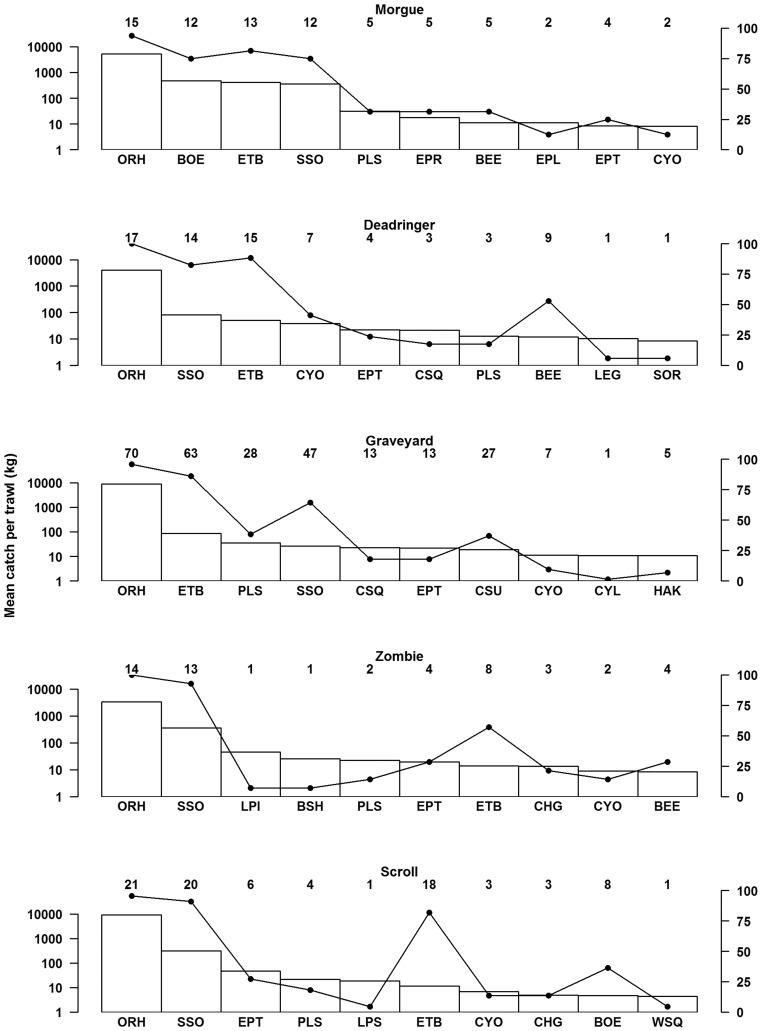
Mean catch rate (bars, log-scale, left axis) and percentage occurrence (line graph, right axis) for the 10 species with the highest catch rates on each seamount. Numbers above each bar represent the number of trawls in which the species was caught. See Appendix S1 for the 3-letter Ministry of Fisheries species codes on the x axes.

The physical data for each seamount were extracted from the NIWA “Seamounts” database [24]. Temperature records were derived from the CSIRO Atlas of Regional Seas [25], a digital atlas of seasonal ocean water properties covering seas around Australia and New Zealand.

**Table 3:** Species found on all five seamounts, on four out of five, and so on, to those found on only one seamount.

No. of seamounts	No. of species found by seamount(s)	Species
5	15	BEE, CHG, CMA, CSQ, CSU, CYO, CYP, EPL, EPT, ETB, HOK, ORH, PLS, SMC, SSO
4	10	APR, BOE, CIN, CSE, EPR, HJO, MCA, RCH, SBI, SND
3	7	CKA, CKX, JAV, LCH, SBK, TSQ, WSQ
2	11	**BCR, BSH, CHP, DWO, LPI, LPS, NNA, RUD, SOR, SSM, WHX**
1	26	**BJA, BTA, CBA, CFA, CHA, CMX, COL, CXH, CYL, GRC, GSP, HAK, HCO, HYP, LEG, MRQ, PDG, PSK, SNE, SUS, TRS, TUB, VCO, VSQ, WHR, WOE**

The rarer species found on only one or two seamounts are highlighted and described in more detail in Table 4. See Appendix S1 for species codes.

**Table 4:** Total number of species and number of rare species found on each seamount.

Seamount	Total No. of species	No. of rarespecies	No. of research tows	Rarer species
Morgue	33	7	16	BCR, CMX, COL, LPS, NNA, SOR, VCO
Deadringer	42	11	17	CHP, GSP, LEG, LPI, RUD, SOR, SSM, SUS, TRS, TUB, WHR
Graveyard	51	20	73	BJA, BSH, BTA, CBA, CFA, CHA, CHP, CYL, DWO, GRC, HAK, HCO, NNA, PDG, PSK, RUD, SSM, VSQ, WHX, WOE
Zombie	27	5	14	BCR, BSH, HYP, LPI, MRQ
Scroll	31	5	22	CXH, DWO, LPS, SNE, WHX

See Appendix S1 for 3-letter species codes.

### Analysis

#### Species dominance

Species dominance was determined by the mean catch rate, defined as catch per tow (kg). Catch rates were not divided by tow length or swept area of the trawl net as some tows on the smaller seamounts had very short tow lengths, and gear performance when towing down a rough-sided seamount is unlikely to be constant.

#### Species richness

Total species richness on each seamount was estimated by fitting a “species accumulation curve”, as described by Colwell and Coddington [26], to the survey catch data by calculating the total number of species caught after each successive trawl from the first trawl in the first survey to the last trawl in the last survey, using non-linear least squares estimation. The species accumulation curve *S*(*n*) represents the expected number of species found in *n* stations, and takes the hyperbolic form.
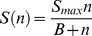



The 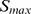 parameter is the asymptote of the curve representing the estimated number of species that would theoretically be found if a very large number of stations were completed, and B is a curvature parameter.

Mean species richness (i.e., average number of species caught in a single tow) is also calculated for each seamount. This is a measure of the diversity which can be expected within a single sample, as opposed to the diversity which would be found over many samples.

**Figure 3:**
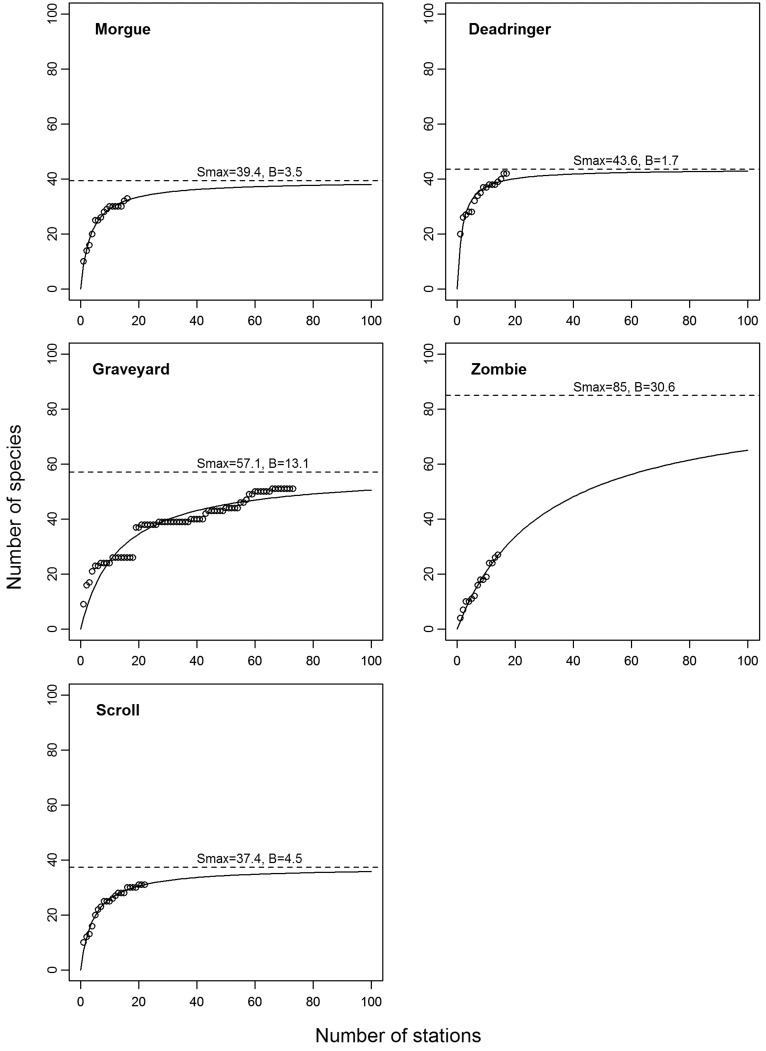
Diversity curve (potential number of species caught with number of stations) and the total species richness (asymptote curve) for each seamount. The symbols on each plot represent actual number of species recorded after the number of trawls indicated.

**Table 5:** Species richness (mean number of species per km trawled) by seamount.

Seamount	Mean species richness (per km)
Morgue	20.7
Deadringer	7.4
Graveyard	20.0
Zombie	19.5
Scroll	26.5

**Table 6:** Species occurring in a significantly greater proportion of tows on Seamount1 than on Seamount2, based on Fisher’s exact test with a 99% significance level.

Species	Seamount1	Seamount2
BOE	Morgue	Graveyard
BOE	Morgue	Zombie
BOE	Graveyard	Zombie
BOE	Deadringer	Zombie
SBI	Deadringer	Graveyard
CYO	Deadringer	Graveyard
EPR	Morgue	Graveyard

**Table 7:** Pair-wise similarity (Ppos) between species for each pair of seamounts.

	Deadringer	Graveyard	Morgue	Scroll	Zombie	Mean similarity
Deadringer	1.00	**0.71**	0.69	0.68	0.67	0.69
Graveyard	**0.71**	1.00	0.62	0.66	0.59	0.65
Morgue	0.69	0.62	1.00	**0.75**	0.57	0.66
Scroll	0.68	0.66	**0.75**	1.00	0.62	0.68
Zombie	0.67	0.59	0.57	0.62	1.00	0.61

Values close to 1 denote strong similarity. Mean similarity shown in the final column is an average of all Ppos seamount values.

#### Frequency of occurrence

Species were also ranked in terms of occurrence on seamount, from those occurring on all five seamounts to those occurring on only one seamount. The gradient from widespread species that occur on all seamounts to rare species which occur only on a few, was investigated.

The significance of differences in species occurrence between seamounts was determined using Fisher’s exact test, based on presence/absence. The 99% significance level was used throughout, to compensate for multiple significance testing.

**Figure 4:**
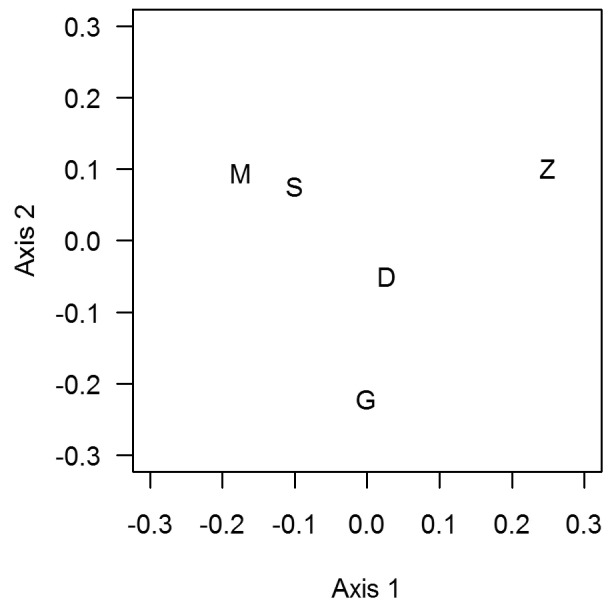
Multidimensional scaling plot of similarity between the species lists of seamounts. Seamounts with the most similar species composition are plotted close together. D, Deadringer; G, Graveyard; M, Morgue; S, Scroll; Z, Zombie.

**Table 8:** Rotating axes coefficients of physical variables from Principal component analysis. PC1 and PC2 are important axes.

	PC1	PC2
**Base depth**	−0.013	**0.796**
Summit depth	0.072	0.242
**Elevation**	−0.085	**0.553**
Area (km^2^)	−0.002	0.003
Slope	0.000	0.020
**Effort**	**−0.994**	−0.040
Cumulative Proportion	0.993	0.999

The variables in bold are seen as the main environmental influences.

#### Faunal similarity

The similarities between fauna on different seamounts were assessed by comparing species lists for each pair of seamounts. The Ppos statistic [27] which is similar to the Sorenson's Index [28] was used to measure the similarity of each pair of species lists. The Ppos number of species in common on both lists was divided by the average length of the two lists, to provide a relative measure. If there were no species in common then Ppos = 0, and if the species lists were identical then Ppos = 1. The resulting similarity in seamount fauna table was displayed graphically in R [29], using classical metric multidimensional scaling and the principal component analysis of seamount features. This method is similar to that of Venables and Ripley [30], and follows that of [19]. The areas are displayed on a 2-dimensional plot so that areas with high faunal similarity are close together, and less similar areas are further apart.

Differences in species diversity and faunal similarity among the five seamounts were analysed by applying analysis of variance and multiple analysis of variance techniques using seamount features such as slope and base depth, as covariates. Principal component analysis of seamount features was also carried out to reduce the number of covariates used for the analysis of variance and multiple analysis of variance models.

Effects on species cycles impacting abundances or species compositions is poorly known. As such any temporal effect such as year of sampling to explain variability or similarities among seamount communities were not explored.

#### Fishing pressure

The level of accumulated commercial fishing effort prior to the surveys was used as a predictor to investigate the effects of fishing on the seamount fish communities. Commercial catch records show a large number of trawls in the vicinity of these seamounts, but for our purposes seamount trawls were defined as those recorded from a rectangular area around each seamount with a tow duration of less than 30 minutes. Manual assignment of trawls to a seamount also took into account the recorded trawl direction and depth [3].

**Figure 5:**
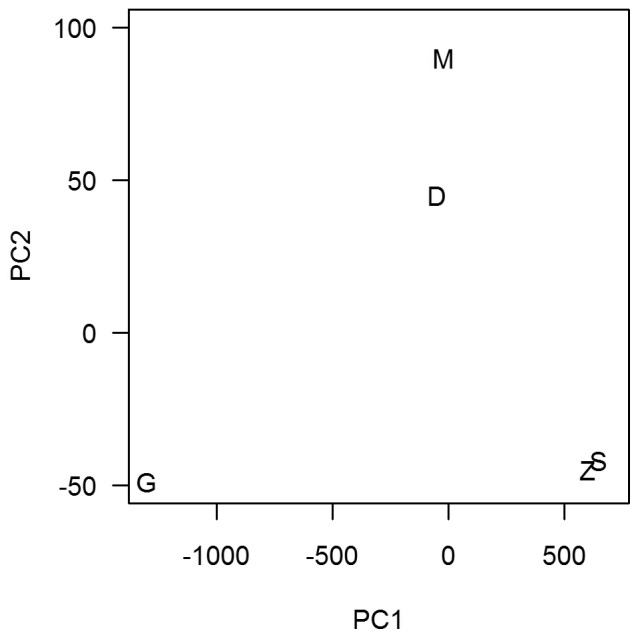
Principal component analysis of seamount features. PC1 is the first principal component 1 and PC2 is the second principal component from Table 8.

**Figure 6:**
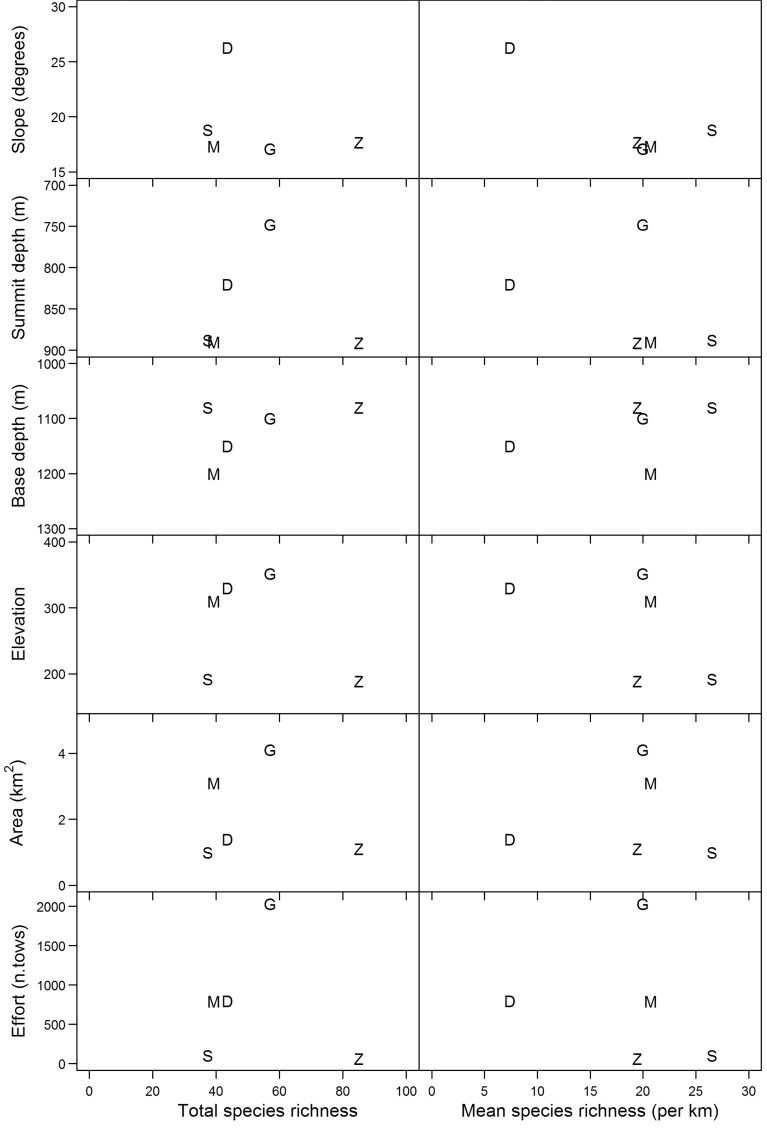
Total species richness and mean species richness plotted against five physical variables (*see* **Table 1**), and commercial fishing effort. D, Deadringer; G, Graveyard; M, Morgue; S, Scroll; Z, Zombie.

**Figure 7:**
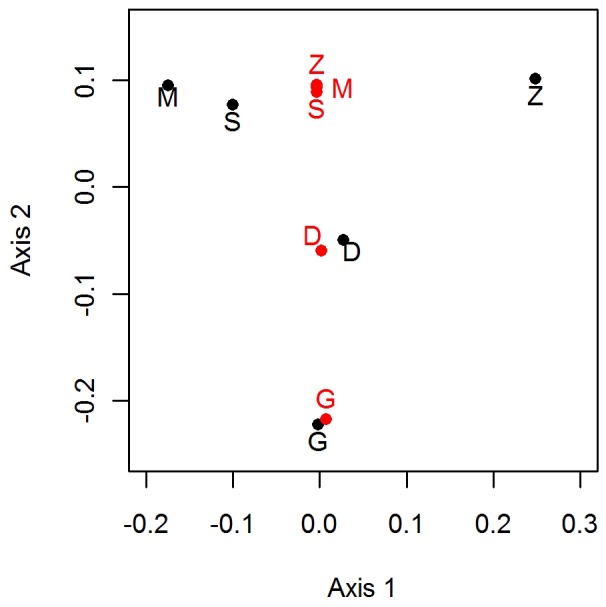
Species similarities between seamounts from Figure 4 (black) and the MANOVA model predictions based on summit depth (red). D, Deadringer; G, Graveyard; M, Morgue; S, Scroll; Z, Zombie.

## Results

### Dominant Species

The ten dominant species at each seamount is shown by descending mean catch rate per trawl in Figure 2. Orange roughy (*Hoplostethus atlanticus*, ORH), was caught in all but five trawls (and in all trawls on Deadringer and Zombie), and produced the highest catch rate on all seamounts. Smooth oreo (*Pseudocyttus maculatus*, SSO) and Baxter’s dogfish (*Etmopterus baxteri*, ETB) were the next most dominant species rating second and third in percentage occurrence on all five seamounts. Other species that were in the top 10 on each feature were two deepsea sharks, plunket shark (*Centroscymnus plunketi*, PLS) and Owston’s dogfish (*Centroscymnus owstoni*, CYO), as well as black cardinalfish (*Epigonus telescopus*, EPT). Black oreo (*Allocyttus niger*, BOE) had a high mean catch rate on Morgue but on all other seamounts this species fell outside the top ten.

### Species Rarity

There were 69 species of fish in total identified from the five seamounts. Table 3 summarises the species common to multiple seamounts, and those found on only a single seamount. Fifteen species were found on all five seamounts, and ten further species were found on four of the five seamounts. Twenty-six species were found on only one seamount.

Seamounts on which the 37 rarer species (those which occurred on only one or two seamounts) were found are shown in Table 4. Rare species are expected to be over-represented on seamounts with many samples compared to seamounts with few tows (as per Clark et al [15], and this is apparent with the results here. Graveyard stands out with the highest number of species, the largest number of rarer species and also is the seamount that has had the greatest number of research tows (as well as being the largest seamount). Deadringer rates second. Deadringer and Morgue have a similar research effort (number of tows) but Deadringer has a higher number of total species and slightly more rare species. Both seamounts are similar in size. Zombie and Scroll produce similar numbers of rare species to Morgue.

Rarer species include the Portuguese dogfish (*Centroscymnus coelolepis*, CYL), lepidion cods (*Lepidion schmidti* and *L. inosimae,* LEG), warty oreo (*Allocyttus verrucosus*, WOE) and macrourid rattails such as black javelin fish (*Mesobius antipodum*, BJA) and humpback rattail (*Coryphaenoides dossenus,* CBA).

### Species Richness

Total species richness is shown in Figure 3 with species accumulation curves presented for each seamount. The model predicts Zombie to have the highest total species richness (85), although this is poorly defined due to the continued capture of new species on each of the relatively few trawls. Graveyard has the next highest species richness (57), partly caused by an unusually large number of new species captured at about the 20^th^ trawl on this seamount. Species richness on the other three seamounts was similar (Scroll, 37; Morgue, 39; Deadringer, 43) and based on a similar number of trawls (16–22).

The mean species richness was considerably lower for Deadringer than for the other four seamounts (Table 5). The values for Morgue, Graveyard, and Zombie were similar and Scroll had the highest level at 26 species per km.

#### Differences in species composition

Pairwise tests showed that in only seven cases was a species significantly more commonly caught on one seamount than on another (Table 6).

Black oreo (BOE) was the second most common species on Morgue (Figure 2), but on both Graveyard and Zombie remained outside of the ten most common species. Black oreo was also more common on Graveyard and Deadringer than on Zombie (none of the 14 trawls on Zombie caught any black oreo). Largescaled brown slickhead (*Alepocephalus australis*, SBI) and Owston’s dogfish (CYO) were both significantly more common on Deadringer than on Graveyard. Although Owston’s dogfish were caught on seven occasions on both Deadringer and Graveyard, there were more than four times as many research trawls on Graveyard. The small cardinalfish (*Epigonus robustus*, EPR) was more common on Morgue (where it was the sixth most common species) than on Graveyard.

The pair-wise similarity (Ppos statistic) results are shown in Table 7. A high similarity (>0.70) occurs between Scroll and Morgue and to a lesser extent between Deadringer and Graveyard. The seamounts least similar in terms of species composition (<0.60) are Zombie and Morgue, and Zombie and Graveyard. Deadringer is more similar to the other four seamounts and Zombie least similar, based on the mean similarity obtained from the average of all Ppos seamount values (excluding the values equal to 1.00).

The patterns of similarity are seen more clearly in a multidimensional scaling plot (Figure 4), where Deadringer is in the centre, Morgue and Scroll are most similar, and Zombie lies more separate.

#### Principal component analysis

Correlations between the physical variables were calculated to avoid using too many similar and highly correlated variables in the model. *Latitude* was more than 95% correlated with *base depth* and *base temp*, as was *longitude* with *slope* (95%), *summit depth* with *peak temp* (100%), and *base depth* with *base temp* (100%). Therefore in the following models the variables *slope*, *base depth*, and *summit depth*, were preferred as they were the most meaningful given the spatial scale and physical characteristics of the five seamounts, along with *effort*, *area*, and *elevation*.

The principal component analysis was carried out using the most meaningful physical variables and they were reduced into two main components with a cumulative proportion of 96.5% (Table 8). *Effort* is important in the first component while the depth variables (particularly *base depth*, and *elevation*) were important in the second component. This analysis shows that it is these two variables that better separate the seamounts.

Seamount base depth (the deepest depth contour which completely encircles the seamount), seamount elevation (the depth range between the seamount summit and base), and fishing effort (the historical number of commercial bottom trawls associated with the seamount) are sufficient to describe the main environmental influences (Table 8). The pseudo-variables PC1 and PC2 are in multiple dimensions (each dimension relating to a real variable), and the influence of each variable on the pseudo-variables is indicated by the values shown in Table 8.

Each seamount feature was examined with multiple physical variables (e.g., elevation, slope, area) and transformed into two principal component axes (Figure 5). From this analysis the spread of points show Scroll and Zombie clustered, but Graveyard, Morgue, and Deadringer positioned well apart as they have different physical features from all other seamounts.

Total and mean species richness in relation to some of the physical characteristics of each seamount, and fishing effort, are compared in Figure 6. In general, total species richness varied between seamounts with different physical characteristics. However, mean species richness was similar for Graveyard, Zombie and Morgue (at about 10 species per km) for each factor. Mean richness on Deadringer was consistently low, and Scroll consistently high.

Analysis of Variance (ANOVA) showed no significant relationship between these features and *total* species richness, but *slope* (*p* = 0.08), was a significant predictor of mean species richness.

#### Species similarity

Species similarity among seamounts was modelled using a multiple analysis of variance (MANOVA) on the values from multidimensional scaling (see axes 1 and 2, Figure 4), and testing each of the physical factors as explanatory variables. Summit depth was the only significant explanatory variable. Summit depth generally explained the species similarity well, especially on Graveyard and Deadringer, but the model did not predict any species differences between Zombie, Morgue, and Scroll (Figure 7).

## Discussion

The study shows clearly that fish species composition and abundance can vary between seamounts, even on a small spatial scale. This was a somewhat surprising result, as many of the common and widespread species found on the seamounts are among New Zealand’s main commercial species ([31], [19], [20]), and most deep-sea fish species in New Zealand waters have a widespread geographical and depth distribution, spanning the ranges of all the seamounts in the study ([32], [21]). Nevertheless, strong differences were apparent, with species like black oreo more common on Morgue than Graveyard, and the species was not recorded at all from Zombie. It was also surprising that a relatively large number of species were recorded from only a single seamount. Partly this may be due to the different sampling effort between the seamounts, with predicted species numbers much greater than actually recorded given the number of tows carried out. This is consistent with the result that rare species were higher in numbers on Graveyard, which had a high number of research station trawls. However, in contrast, Deadringer has had a low number of research trawls but was next highest in numbers of rarer species.

The main environmental factor that was identified as influencing the composition and abundance of the fish communities was depth-both base depth and summit depth. Base depth, elevation, and summit depth are linked, and together describe potential changes in fish species presence due to the actual depths covered by the seamount, and the depth range. Depth is a common factor that has a very strong influence on species composition (e.g., [33], [34]. However, although the MANOVA performed well with Graveyard and Zombie, it was inadequate for the other seamounts, indicating that community composition is more than simply a derivative of depth.

In a larger scale study for the New Zealand region, Tracey et al. (2004) [19] found that total species richness was similar in all seamount regions, but mean species richness much higher in southern areas. A similar latitudinal effect was observed for New Zealand deep-sea fish generally [31], and on the Chatham Rise by Hurst et al. [35]and Beentjes et al. [36]. In the present study, the spatial scale of the seamounts proximity to each other was too small to expect latitude per se to have an influence, but characteristics of slope and summit depth were correlated.

Broad similarities in fish communities over large spatial scales were also recorded by Koslow et al. [37] and Clark et al. [20] with prominent affinities in the species composition between areas as distant as the Southwest Pacific and the north Atlantic Oceans. Prevailing oceanic circulation, and a similar water mass distribution between ocean basins was believed to be the underlying cause of similarities, but Clark et al. [20] also noted strong community patterns characterising only a single locality, and concluded that the distribution of deep-sea species is not a simple relationship with water mass. Although the Graveyard seamounts are all in the same water mass at depth (Antarctic Intermediate Water), the findings of the current research are similar, that environmental factors may affect the distribution of some species, but it is difficult to isolate environmental drivers of overall patterns of assemblage composition.

Total species richness patterns were inconsistent with mean species richness trends. Mean species richness, which avoids the problem of differing sampling effort, was high on Scroll, and low on Deadringer. Reasons for why Scroll was high are unclear. However, it may be significant that Deadringer is the most isolated of the seamounts examined. Graveyard, Morgue, Scroll and Zombie are all within several km of one another, and the cluster of features may provide a greater variety of habitat types and depths to support a larger number of species. Fish association with biogenic habitats, such as outcrops of stony coral, is well documented [38], [39], However, direct linkages between demersal fish and substrate type are not as obvious as for benthic invertebrates, where the type and characteristics of the substratum may control the composition of fauna (e.g., [40], [41], [42]) and high megafaunal diversity can be associated with the variety of topography and microrelief (e.g., [43]. Variation in substrate type between the seamounts is a possible source of confoundment or bias, but all the seamounts in the Graveyard complex have a mixture of substrate [4]. Trawl catches are integrated across patches of rocky and soft sediment seafloor, and together with trawl direction from the summit being randomised on a number of surveys, this reduces the likelihood of any substrate effect.

The level of historical fishing effort was a significant factor affecting community composition when we investigated species similarity with a principal components analysis.

Disturbance to the environment is expected to cause species that tolerate the perturbation to thrive, while intolerant species decline in abundance [44]. Differences in diversity may result from only certain species being targeted by the fishery (in this area primarily orange roughy), but changes can involve both a reduction in diversity as rare species are eliminated, or an increase in species richness by reducing abundance of the dominant species and allowing new species to gain a foothold (e.g., [45], [46], [47]). Differences in life history characteristics (e.g. slower growth, larger size at maturity) also make certain species more vulnerable to fishing, and many seamount-associated species have been classed as highly vulnerable (e.g., [17]). In studies of invertebrate fauna for the region, strong differences in community composition were reported between fished and unfished hills in the Graveyard Complex by Clark and O’Driscoll and Clark and Rowden [8], [3].

Within the North Sea, patterns of diversity of the groundfish assemblage have changed over time, and in areas with different fishing histories. However, changes have varied, with reduced diversity reported in some heavily fished areas (e.g., [48], [49]), while other studies have noted increased diversity in some areas [50], [51]. Bianchi et al. [52] examined a global dataset, and found differences in diversity measures caused either by changes in the patterns of dominance or in the numbers of species because of improved species identification. Off Alaska, reduced fishing effort was thought to be the cause of an increase in the frequency of occurrence of a number of fish species during the 1990s [53].

Comparative biodiversity studies are often difficult to achieve, because of issues with data or analytical techniques. Taxonomic consistency is often a major problem with deep-sea data sets from seamounts where new species are often found (e.g. [13]). The fish identifications used in this study were made by experienced fisheries scientific staff, and where uncertain verified by museum fish taxonomists. Utilising research data also ensured that the species composition of the entire catch was established. Uneven sampling effort was dealt with by comparing asymptotic estimates of total species richness, and by mean richness per tow. Other studies have reduced the uneven sample sizes to a smaller number across all seamounts (e.g., [20]). However, this latter approach does not utilise all the available data, and potentially loses information. Most of the asymptotic predicted diversity curves derived in this study looked reasonable against the actual samples, but Zombie stands out as an anomaly because of the large difference between actual recorded and estimated number. The Zombie result has to be regarded as highly uncertain when interpreting of the differences in diversity levels.

Care is also needed extrapolating species richness to the entire seamount area. The estimated total species richness was highest for Graveyard, which was also the largest seamount with the greatest elevation. The other seamounts generally had similar levels despite differences in size. It is common ecological theory that the number of benthic species increases with area (e.g., [54]) and a larger seamount will generally have more species than a small one because it spans a greater depth range and will have a more varied complex of substrate types and micro-habitats. Depth range can affect demersal fish distribution, but while habitat complexity is definitely relevant for benthic invertebrates, it is uncertain whether it applies equally to fish, especially the larger-bodied species caught by the trawl gear used in this study. The variation in seamount size in this study is probably insufficient to draw firm conclusions about this result.

This study has been able to focus on a unique dataset from a complex of seamounts in close proximity to one another and reasonably isolated from other seamounts. However, the types of fish species caught by bottom trawl gear with heavy bobbin and rockhopper ground gear tend to be larger species, or larger sized fish. Catches may not, therefore, represent the full fish community, or very small spatial scale structure which may be associated with variable habitats within a seamount (e.g., [55]). Such studies ideally should incorporate other methods as well, such as data from ROV’s and towed camera systems. Recent research has shown that the effects of climate change will extend into deep sea environments and stress the need to increase the collection of seamount specific environmental data sets to help improve these types of analyses.

## Supporting Information

Appendix S1 **Species included in the analysis and their composition by seamount in the Graveyard Complex.** Species are identified by unique 3-letter Ministry of Fisheries species codes. D, Deadringer; G, Graveyard; M, Morgue; S, Scroll; Z, Zombie.
(DOCX)
