# Supplementary material for: Deep-Sea Fish Distribution Varies between Seamounts: Results from a Seamount Complex off New Zealand
Source: PLoS One. 2012 Jun 20;7(6):e36897. doi: 10.1371/journal.pone.0036897 (PMC3380042; doi:10.1371/journal.pone.0036897)
Supplement: Appendix S1 — Species included in the analysis and their composition by seamount in the Graveyard Complex. Species are identified by unique 3-letter Ministry of Fisheries species codes. D, Deadringer; G, Graveyard; M, Morgue; S, Scroll; Z, Zombie. (DOCX) [file pone.0036897.s001.docx]

Appendix S1: Species included in the analysis and their composition by seamount in the Graveyard Complex. Species are identified by unique 3-letter Ministry of Fisheries species codes. D, Deadringer; G, Graveyard; M, Morgue; S, Scroll; Z, Zombie.

| Species code | Common name | Scientific name | D | G | M | S | Z |
| --- | --- | --- | --- | --- | --- | --- | --- |
| APR | Catshark | *Apristurus* spp. | * | * | * | * |  |
| BCR | Blue cusk eel | *Brotulotaenia crassa* |  |  | * |  | * |
| BEE | Basketwork eel | *Diastobranchus capensis* | * | * | * | * | * |
| BJA | Black javelin fish | *Mesobius antipodum* |  | * |  |  |  |
| BOE | Black oreo | *Allocyttus niger* | * | * | * | * |  |
| BSH | Seal shark | *Dalatias licha* |  | * |  |  | * |
| BTA | Smooth deepsea skate | *Brochiraja asperula* |  | * |  |  |  |
| CBA | Humpback rattail | *Coryphaenoides dossenus* |  | * |  |  |  |
| CFA | Banded rattail | *Coelorinchus fasciatus* |  | * |  |  |  |
| CHA | Viper fish | *Chauliodus sloani* |  | * |  |  |  |
| CHG | Giant chimaera | *Chimaera lignaria* | * | * | * | * | * |
| CHP | Brown chimaera | *Chimaera sp.C* | * | * |  |  |  |
| CIN | Notable rattail | *Coelorinchus innotabilis* | * | * | * | * |  |
| CKA | Kaiyomaru rattail | *Coelorinchus kaiyomaru* | * | * | * |  |  |
| CKX | Spottyfaced rattails | *Coelorinchus* spp*.* | * | * |  |  | * |
| CMA | Mahia rattail | *Coelorinchus matamua* | * | * | * | * | * |
| CMX | McMillan's rattail | *Coryphaenoides mcmillani* |  |  | * |  |  |
| COL | Oliver’s rattail | *Coelorinchus oliverianus* |  |  | * |  |  |
| CSE | Serrulate rattail | *Coryphaenoides serrulatus* | * | * | * | * |  |
| CSQ | Leafscale gulper shark | *Centrophorus squamosus* | * | * | * | * | * |
| CSU | Four-rayed rattail | *Coryphaenoides subserrulatus* | * | * | * | * | * |
| CXH | Horrible rattail | *Coelorinchus horribilis* |  |  |  | * |  |
| CYL | Portuguese dogfish | *Centroscymnus coelolepis* |  | * |  |  |  |
| CYO | Owston’s dogfish | *Centroscymnus owstoni* | * | * | * | * | * |
| CYP | Longnose velvet dogfish | *Centroscymnus crepidater* | * | * | * | * | * |
| DWO | Deepwater octopus | *Graneledone* spp*.* |  | * |  | * |  |
| EPL | Bigeye cardinalfish | *Epigonus lenimen* | * | * | * | * | * |
| EPR | Robust cardinalfish | *Epigonus robustus* | * | * | * | * |  |
| EPT | Deepsea cardinalfish | *Epigonus telescopus* | * | * | * | * | * |
| ETB | Baxter’s lantern dogfish | *Etmopterus baxteri* | * | * | * | * | * |
| GRC | Grenadier cod | *Tripterophycis gilchristi* |  | * |  |  |  |
| GSP | Pale ghost shark | *Hydrolagus bemisi* | * |  |  |  |  |
| HAK | Hake | *Merluccius australis* |  | * |  |  |  |
| HCO | Hairy conger | *Bassanago hirsutus* |  | * |  |  |  |
| HJO | Johnson's cod | *Halargyreus johnsonii* | * | * |  | * | * |
| HOK | Hoki | *Macruronus novaezelandiae* | * | * | * | * | * |
| HYP | Pointynose blue ghost shark | *Hydrolagus trolli* |  |  |  |  | * |
| JAV | Javelin fish | *Lepidorhynchus denticulatus* |  | * | * | * |  |
| LCH | Longnose spookfish | *Harriotta raleighana* | * | * |  |  | * |
| LEG | Deepsea lepidion cods | *Lepidion schmidti* & *L.inosimae* | * |  |  |  |  |
| LPI | Round tooth lepidion | *Lepidion inosimae* | * |  |  |  | * |
| LPS | Giant lepidion | *Lepidion schmidti* |  |  | * | * |  |
| MCA | Ridge scaled rattail | *Macrourus carinatus* | * | * |  | * | * |
| MRQ | Warty squid | *Onykia robsoni* |  |  |  |  | * |
| NNA | Nezumia rattail | *Nezumia namatahi* |  | * | * |  |  |
| ORH | Orange roughy | *Hoplostethus atlanticus* | * | * | * | * | * |
| PDG | Prickly dogfish | *Oxynotus bruniensis* |  | * |  |  |  |
| PLS | Plunket’s shark | *Centroscymnus plunketi* | * | * | * | * | * |
| PSK | Longnosed deepsea skate | *Bathyraja shuntovi* |  | * |  |  |  |
| RCH | Pacific spookfish | *Rhinochimaera pacifica* | * | * | * |  | * |
| RUD | Rudderfish | *Centrolophus niger* | * | * |  |  |  |
| SBI | Bigscaled brown slickhead | *Alepocephalus* sp. | * | * |  | * | * |
| SBK | Spineback | *Notacanthus sexspinis* | * | * | * |  |  |
| SMC | Small-headed cod | *Lepidion microcephalus* | * | * | * | * | * |
| SND | Shovelnose dogfish | *Deania calcea* | * | * | * | * |  |
| SNE | Snubnosed eel | *Simenchelys parasiticus* |  |  |  | * |  |
| SOR | Spiky oreo | *Neocyttus rhomboidalis* | * |  | * |  |  |
| SSM | Smallscaled brown slickhead | *Alepocephalus australis* | * | * |  |  |  |
| SSO | Smooth oreo | *Pseudocyttus maculatus* | * | * | * | * | * |
| SUS | Centrolophid medusafish | *Schedophilus* sp. | * |  |  |  |  |
| TRS | Cape scorpionfish | *Trachyscorpia capensis* | * |  |  |  |  |
| TSQ | Todarodes squid | *Todarodes filippovae* | * | * |  |  | * |
| TUB | Tasmanian ruffe | *Tubbia tasmanica* | * |  |  |  |  |
| VCO | Violet cod | *Antimora rostrata* |  |  | * |  |  |
| VSQ | Violet squids | *Histioteuthis* spp. |  | * |  |  |  |
| WHR | Unicorn rattail | *Trachyrincus longirostris* | * |  |  |  |  |
| WHX | White rattail | *Trachyrincus aphyodes* |  | * |  | * |  |
| WOE | Warty oreo | *Allocyttus verrucosus* |  | * |  |  |  |
| WSQ | Warty squids | *Onykia* spp. | * |  | * | * |  |
